# Does Masculinity Matter? The Contribution of Masculine Face Shape to Male Attractiveness in Humans

**DOI:** 10.1371/journal.pone.0013585

**Published:** 2010-10-27

**Authors:** Isabel M. L. Scott, Nicholas Pound, Ian D. Stephen, Andrew P. Clark, Ian S. Penton-Voak

**Affiliations:** 1 School of Experimental Psychology, University of Bristol, Bristol, United Kingdom; 2 Department of Psychology, Brunel University, Uxbridge, United Kingdom; Indiana University, United States of America

## Abstract

**Background:**

In many animals, exaggerated sex-typical male traits are preferred by females, and may be a signal of both past and current disease resistance. The proposal that the same is true in humans – i.e., that masculine men are immunocompetent and attractive – underpins a large literature on facial masculinity preferences. Recently, theoretical models have suggested that current condition may be a better index of mate value than past immunocompetence. This is particularly likely in populations where pathogenic fluctuation is fast relative to host life history. As life history is slow in humans, there is reason to expect that, among humans, condition-dependent traits might contribute more to attractiveness than relatively stable traits such as masculinity. To date, however, there has been little rigorous assessment of whether, in the presence of variation in other cues, masculinity predicts attractiveness or not.

**Methodology/Principal Findings:**

The relationship between masculinity and attractiveness was assessed in two samples of male faces. Most previous research has assessed masculinity either with subjective ratings or with simple anatomical measures. Here, we used geometric morphometric techniques to assess facial masculinity, generating a morphological masculinity measure based on a discriminant function that correctly classified >96% faces as male or female. When assessed using this measure, there was no relationship between morphological masculinity and rated attractiveness. In contrast, skin colour – a fluctuating, condition-dependent cue – was a significant predictor of attractiveness.

**Conclusions/Significance:**

These findings suggest that facial morphological masculinity may contribute less to men's attractiveness than previously assumed. Our results are consistent with the hypothesis that current condition is more relevant to male mate value than past disease resistance, and hence that temporally fluctuating traits (such as colour) contribute more to male attractiveness than stable cues of sexual dimorphism.

## Introduction

Many researchers studying non-human mate choice have observed that exaggerated sex-typical male traits, such as large antlers and peacock's tails, are attractive to females [1]. Authors have suggested that the growth of such traits is mediated by immune-stressing steroids such as testosterone, and that as only high quality males can “afford” exposure to immune stress, these traits signal high levels of immunocompetence [2]–[7]. Such perspectives have generated similar expectations regarding human mate choice – i.e. that masculine males should be attractive, and that this attractiveness is attributable to immunocompetence [8]. These proposals form the basis of a large literature on human preferences for facial masculinity [9].

More recently however, a number of authors have questioned immunocompetence perspectives on facial masculinity preferences. Recent reviews of the animal literature present a complex and uncertain picture of the relationship between immunity, testosterone and trait size [7], [10]. In humans, preliminary evidence suggests there is an association between circulating testosterone levels and anatomical masculinity in faces [11], but the evidence for an association between either testosterone or masculinity and disease resistance is scant, inconsistent, and largely negative [7], [12]–[20]. Even if masculinity *does* signal past disease resistance, it is unclear that females will, in general, benefit from attending to this signal, particularly if cues to current condition are available. Past disease resistance may be a weak predictor of current and future resistance, especially if pathogenic complexity is high, and pathogen fluctuation is fast (relative to host lifespan and generation length) [21], [22]. Recent mathematical models of mate choice suggest that in most environments, females can reliably derive substantial fitness advantages from attending to current condition, but may gain little, if any, further benefit from simultaneously selecting mates on the basis of past immune function [21], [22]. Thus, stable traits such as masculinity, which are not influenced by short-term fluctuations in adult health, should be of less importance to attractiveness than other more condition-responsive cues. This expectation is stronger in animals with long lifespans and slow reproduction, such as humans.

Consistent with this reasoning, findings relating male attractiveness to long-term health and/or stable facial traits have to date been equivocal [14], [23]–[27]. In particular, reported masculinity preferences have been highly inconsistent across participants and methodologies – in stark contrast to men's reliable preferences for facial femininity in women [9], [20], [28]–[30]. In spite of this, and of the large volume of literature on masculinity preferences, little attempt has been made to quantify the contribution of naturally-occurring variations in facial masculinity to “real life” attractiveness. Many studies to date have employed computer-based morphing methods to increase or decrease the masculinity of a particular facial photograph, and thereby measure the influence of masculinity on preferences. As such methods eliminate variation in other, potentially competing cues to attractiveness, they force participants (often in a forced-choice paradigm) to attend to masculinity alone, and cannot be used to gauge its importance in realistic contexts. While correlational approaches using unmodified photographs of individuals should address this concern, experiments to date have largely relied on subjective measures (i.e. ratings) of facial masculinity as independent variables [18], [31]–[34]. Few studies have attempted objective assessments of anatomical masculinity in faces, and those that have done so have used relatively simple measures and/or produced inconclusive results [14], [27], [31], [35].Using the best measure (of which we are aware) of facial masculinity to date, researchers found no evidence of a relationship between masculinity and attractiveness, although this measure correctly classified only 75% of faces by sex [14]. The importance of facial sexual dimorphism as a component of attractiveness is therefore, surprisingly, currently unknown.

In contrast to the large body of literature regarding the role of stable traits in human mate choice [9], research on condition and attractiveness has been limited. In spite of this, empirical evidence is broadly consistent with the view that men's current health influences attractiveness, [9], [34], [36]–[38,although see 39], and a number of cues have been identified via which this influence may be achieved. Skin cues such as overall skin colour and colour homogeneity, for example, are observable, objectively measurable, and known correlates of condition in humans and non-human animals [38], [40], [41]. Colour information influences judgments of attractiveness [40], [42], health [41]–[43] and facial identity [44], and may contribute more to sex-discrimination than does shape information [45], [46]. Research on attractiveness and skin colour is a relatively recent phenomenon however, and as with masculinity research, has largely relied on subjective measures or morphing techniques [38], [41]. Those studies that have used objective measures of natural variation in skin colour, and tested whether they predict attractiveness in the presence of variation in competing cues, have been limited to female faces [40], [42]. It is unclear, therefore, whether skin colour is an important component of male attractiveness.

To explore these issues, we measured associations between sexual dimorphism and attractiveness in male faces. In two independent samples, geometric morphometric analysis of the configuration of a large number of facial landmarks was used to generate an objective measure of natural variation in morphological masculinity, and the extent to which it predicted attractiveness was assessed. To further investigate the relative contribution of stable versus condition-dependent cues, we extracted facial skin colour information from the faces. This information was entered into a regression model along with morphometric masculinity to determine the extent to which either one could predict attractiveness.

## Methods

Experiments were conducted using two photo-samples.

### Participants

Subjects participated in the ratings experiment in exchange for course credit or cash payment.

#### Sample 1

Twenty-two female undergraduate students (age range 18–21, mean 19.5, SD .66), recruited via University of Bristol.

#### Sample 2

Forty-nine students and members of staff from Bristol University. Eighteen [10 women, 8 men, age range 19–41, mean age 27, SD 7.3] viewed whole faces. Thirty-one (20 women, 11 men, age range 18–70, mean age 31, SD 11) viewed skin patches only.

### Stimuli

Two sets of colour facial photographs of Caucasian males who were facing forward, and told to adopt a neutral , relaxed expression were employed in this study.

#### Sample 1

Twenty photos collected from a community sample of men from northern England (mean age 27, SD = 3). Participants were photographed sitting, 1.5 metres from a digital camera (Nikon E950) in front of a black background. Subjects were illuminated with fluorescent light with no flash.

#### Sample 2

Seventy-five photos collected from students at Stirling University (mean age 21, SD = 2). Skin patch stimuli were also generated from these photos (section 2.3.2). Participants were standing (ensuring replicable natural head position), 1.5 metres from the digital camera (Canon PowerShot G1), in front of a grey background. Subjects were lit with bilateral studio lights (slightly offset to provide some depth information), in a room with no natural light. No flash was used.

### Measures

#### Morphometric masculinity: Sample 1

The 20 male faces were part of a larger photoset of 62 male and female faces from the same population of adults. A geometric morphometric analysis of all of these faces was used to generate morphological masculinity scores for each face in a manner analogous to that use used for previously for bodies [47]. First, using criteria established by Stephan et al [48], the x-y coordinates of 129 facial landmarks (Fig. S1 – supplementary material) were delineated for each face using Psychomorph [49]. Geometric morphometric techniques were then used to calculate a masculinity index for each face. Morphologika [50] was used to carry out Procrustes registration of the landmark data - a best fit procedure that removes scale, rotational and translational differences between shapes [51]–[53].

Next, to identify dimensions of variation in facial landmark configuration, Morphologika was used to conduct Principle Components Analysis (PCA) of the Procrustes-registered landmark data. A Kaiser-Guttman criterion was used to select Principle Components (PCs) for inclusion in subsequent analysis; i.e. those with eigenvalues greater than the average eigenvalue were retained. This led to the retention of the first 11 PCs which together accounted for 84.7% of the variance in facial landmark configuration (see Table S1, supplementary material for details).

Step-wise discriminant analysis (SPSS 13) was then used to establish which of the 11 PCs were best able to discriminate between the male and female faces. The resulting discriminant function incorporated eight of the PCs (Wilks' λ = 0.163; df = 8; χ2 = 101.6, p<0.00001), and yielded correct sex classifications for 96.8% of faces (see Table S1, and Fig. S2, supplementary material, for details). Discriminant function scores were therefore used as an index of morphological masculinity, with high scores indicating a more masculine facial structure (see Table S1, supplementary material for details).

#### Morphometric masculinity: Sample 2

**Figure 1:**
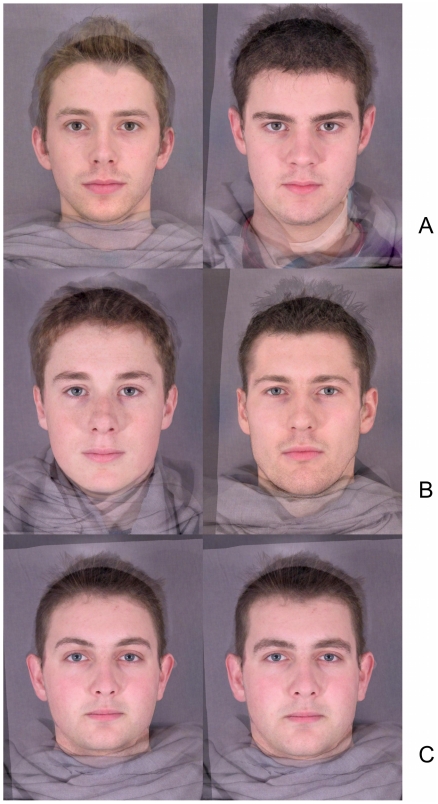
Masculinity, measured, rated and morphed: examples of dimensions. To preserve anonymity of participants, these faces are composites rather than real individuals. a) Morphological masculinity. Examples of faces scoring low (left) and high (right) on this measure. b) Rated masculinity. Examples of faces rated as low (left) and high (right) masculinity. c) Digitally morphed masculinity. Example of a face morphed in the feminine (left) and masculine (right) direction.

Morphological masculinity was calculated in the same manner as sample 1, using a set of 150 faces (75 male, 75 female) from the same population, and with discriminant function scores again being used as an index of facial masculinity (with high scores indicating a more masculine facial structure; Fig. 1 a) for examples). Twenty-one PCs were retained from the PCA, accounting for 85.7% of the variance in facial landmark configuration (Table S2). Step-wise discriminant analysis determined that 11 PCs were best able to discriminate between the male and female faces. The resulting discriminant function was again a powerful discriminator (Wilks' *λ* = 0.134; *df* = 11; *χ*2 = 286.6, *p*<0.00001), yielding correct sex classifications for 98.7% of participants (Table S2, Fig. S3).

#### Skin colour

**Figure 2:**
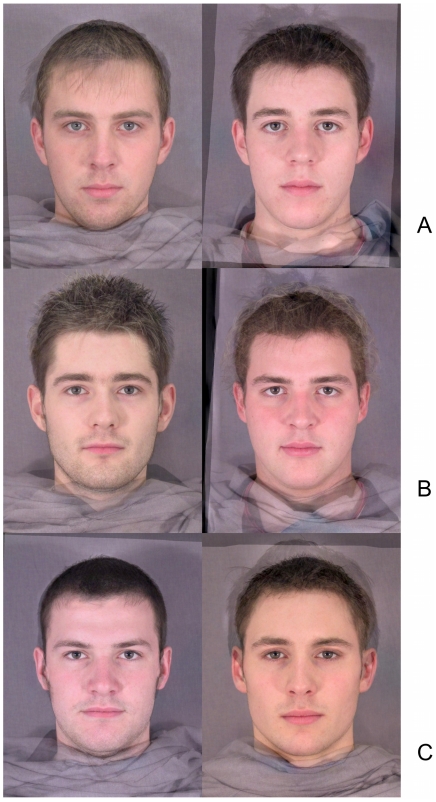
Colour variation: examples of stimuli. a) Faces scoring low (left) and high (right) for lightness (L*). b) Faces scoring low (left) and high (right) for redness (a*). c) Faces scoring low (left) and high (right) for yellowness (b*).

For each face from sample 2, the average lightness, redness and yellowness, as defined by the CIELab color space, was calculated across pixels using Matlab (see Fig. 2, for examples). The CIELab color space is defined by L* (lightness), a* (redness) and b* (yellowness) color dimensions, and is modelled on the human visual system. It is designed to be perceptually uniform, with a change of one unit appearing to be of approximately the same magnitude regardless of its dimension [54]. Increases in facial skin L*, a* and b* values enhance apparent health[41], [43] and therefore affect attractiveness in human faces [38].

In order to check whether skin appearance itself – and not just some morphological correlate of skin appearance – contributes to attractiveness, we also collected ratings of skin health using stimuli that did not display shape information. To create the stimuli, patches of 114*142 pixels were extracted from both left and right cheeks of the faces from sample 2, with the inner top corner of each patch positioned at a fixed height vertically below the pupil. The resulting skin patch stimuli displayed colour but not shape information (see supplementary material, Fig. S4, for examples), and were used to examine the relationship between objective colour cues, perceptions of skin health, and whole face attractiveness. To this end, skin patches were rated for apparent health by 31 independent participants, following prior methods [38]. Each image was enlarged by 100% and then presented to participants in a random order on a computer screen, who rated them for health on a scale of 1 to 7. Inter-rater reliabilities were high (Cronbach's alpha  = .950 (left patches), .963 (right), .954 (both sides together)). For each face, ratings were averaged for left and right patches across all participants to create an overall skin health score.

#### Rated attractiveness: Sample 1

Subjects viewed a computer presentation of the 20 male photos, in random order, and rated each of them for attractiveness on a scale of 0–9. Each photograph was assigned a score for rated attractiveness by averaging responses across participants. Inter-rater reliability was high (Cronbach's α = .894).

#### Rated attractiveness: Sample 2

Photographs were rated in the same manner as sample 1, using a scale of 1–7. Inter-rater reliability was high (Cronbach's α = .932). To determine whether any patterns were specific to male faces, the 75 female faces were also rated for attractiveness. Inter-rater reliability was again high (Cronbach's α = .897).

## Results and Discussion

Kolmogorov-Smirnov tests indicated that all measures (attractiveness, morphological masculinity, skin redness, skin yellowness, skin-patch health ratings) were normally distributed within each sample of males. Sample 2 contained one outlier for morphological masculinity and two for skin redness; that is, cases with values that were more than 1.5 times the interquartile range higher than the third quartile or lower than the first quartile. Outliers are excluded from the relevant subsequent analyses, although including them did not affect the significance of results.

### Morphological masculinity and attractiveness

Linear regressions were conducted, with faces as subjects, morphological masculinity as the independent variable and mean attractiveness rating for each face as the dependent. There was no significant linear relationship between these two variables in either set of male photographs (sample 1: F(1,19) = 1.134, beta = .243, t = 1.065, p = .301, sample 2: F(1,73)  = 1.108, beta = .123, t = 1.053, p = .296). Including further nonlinear terms indicated likewise that the relationship between morphological masculinity and attractiveness was not significantly approximated by a quadratic or logarithmic model (sample 1: both F<1.36, p>.259, sample 2: both F<1.024, p>.315).

To validate our measure of morphological masculinity, analyses were repeated for the female faces from sample 2 (see section 2.3.2). In contrast to the male faces, the relationship between masculinity and attractiveness in female faces was significant and negative (F(1,72^†^) = 6.339, beta = −.286, t = 2.518, p = .014, ^†^two outliers were excluded from analyses), implying that the most feminine (i.e. least masculine) females were attractive, and that our measures would have detected a similar effect in male faces had it existed.

To exclude the possibility that our null findings were due to individual variation in female preferences cancelling each other out (as a result of menstrual cycle phase, own attractiveness or any other individual difference variable that has been shown to influence preferences for masculinity in face shape), additional correlations between morphological masculinity and attractiveness ratings were performed for each participant individually. In sample 1, significant correlations between morphological masculinity and attractiveness ratings were observed among two of the participants (both correlations positive, p<.05), and in sample 2 there were no significant correlations (all p>.05). Thus, among the large majority (95%) of raters, there was no evidence of preference for masculinity, either positive or negative, a finding consistent with a generalised indifference to masculinity as a cue of mate value.

### Skin colour and attractiveness

To investigate the effect of skin colour, a further (backward) linear regression was conducted on the faces from sample 2, with attractiveness as a dependent variable, and morphological masculinity, skin lightness, yellowness, and redness and as independent variables. There was one significant correlation among the independent variables (between skin yellowness and skin lightness (Pearson correlation r(75) = −.315, p<.01)), but tolerance testing indicated that standard assumptions regarding multicolinearity were not violated (all VIF<1.141). The regression retained only skin yellowness as a predictor of attractiveness, and the effect of skin yellowness was positive and highly significant (F(1,71) = 10.806, Beta = .366, t = 3.287, p<.002). Skin lightness, redness and morphological masculinity did not significantly predict attractiveness (all p>.114, see Table 1).

**Table 1:** Relationship between objective traits and attractiveness.

	*Sample 1 (n = 20)*		*Sample 2 (n = 74* † *), masculinity only in model*		*Sample 2 (n = 72* † *), masculinity and colour cues in same model*	
	Association with Attractiveness^a^					
*Independent variables*	Beta	p	Beta	p	Beta	p
Morphological masculinity^b^	.243	.301	.123	.296	.140	.215
Skin lightness	-	-	-	-	−.185	.114
Skin redness^b^	-	-	-	-	.011	.921
Skin yellowness^b^	-	-	-	-	.366	.002**

Result of linear regressions with attractiveness as dependent variable, and morphological masculinity and skin colour as independent variables.

a As rated by participants.

b Measured using methods described in section 2.3.

† Total sample  = 75, outliers excluded where appropriate.

**p<.01.

While skin cues were therefore correlated with attractiveness, it is feasible that participants' responses to faces were not actually influenced by skin appearance, and instead were determined exclusively by some non-dimorphic shape cue, which was also a correlate of skin appearance. To explore this possibility, ratings of skin-patch health were entered into a Pearson correlation with whole-face attractiveness ratings. Results showed a significant correlation between rated skin-patch health and whole-face attractiveness (r(75) = .266, p = .021). As the skin patches did not contain any visual information regarding shape/morphology, this relationship suggests that responses to faces are influenced (at least in part) by skin appearance, and not entirely by shape-correlates of skin colour. It is therefore likely that skin appearance actually affects attractiveness, rather than merely being associated with it in virtue of some other third variable.

There was no evidence of a main effect of masculinity on attractiveness, but masculinity may nevertheless be a “second- pass” predictor of attractiveness. That is, masculinity may be attractive in faces which exhibit other, more important cues of attractiveness, and irrelevant in other faces. This would make it more difficult to detect a main effect of masculinity on attractiveness. To examine this possibility, we performed a moderator analysis [55], with skin yellowness treated as a potential moderator of the influence of masculinity on attractiveness. That is, a hierarchical multiple regression was performed, with morphological masculinity and skin yellowness (both z-standardised) entered in step one, and the product of these two variables entered in step two. Results indicated that only skin yellowness was a significant predictor of attractiveness in this model (Beta = .379, t = 3.418, p = .001). Neither masculinity nor the interaction between masculinity and skin yellowness were significant predictors of attractiveness (both Beta<.15, p>217). There was therefore no evidence in our data that masculinity was either a first or a second-pass criterion of attractiveness.

### The influence of testing methodology

Our results suggest that women are indifferent to morphological masculinity when viewing unmanipulated faces of individual men. A number of previous authors have reported that women do respond to male facial masculinity when making judgments about attractiveness. However, this apparent contradiction may be attributable to some important methodological differences between the present study and previous work.

As outlined earlier, most previous research in this area has depended on either examining associations between attractiveness ratings and subjective measures of *perceived* masculinity for individual faces; or using morphing techniques to create stimuli based on male-female differences in face shape for attractiveness rating/choice tasks. The subjective approach has generally found a small, but consistently positive association between *perceived* masculinity and attractiveness. However, the use of subjective measures is problematic; ratings of masculinity are unlikely to be based on judgements of face shape alone, and the term “masculinity” is liable to being interpreted as normative, and therefore to imply health and/or attractiveness. Consistent with this proposal, prior authors have found that rated masculinity is correlated with perceived health, and that this may explain part of the attractiveness of masculine-rated faces [34]. Associations between rated masculinity and attractiveness may not, therefore, imply a relationship between objective shape-masculinity and attractiveness.

Morphing methods often use objective criteria of masculinity, but are subject to the alternative shortcoming that only one variable is manipulated in the construction of the stimuli, and choice based on other variation in facial appearance is eliminated. As a result, such experiments offer little information about the contribution of masculinity to attractiveness in real faces, where other, potentially more salient traits will also vary, and may eclipse any variation in masculinity. Indeed, recent research suggests that other shape traits which influence responses in morphing experiments may explain very little variation in the attractiveness of real faces [26], [56]. Moreover, so long as the female participants share a common, systematic method of ranking the faces viewed in such experiments, test responses will be non-random – *even if participants are indifferent to masculinity*. Systematic biases in tests for masculinity preferences might, for example, be an epiphenomenon of preferences for averageness; averageness preferences are widely documented, and masculinising a face makes it either more or less like the male average, depending on its starting level of masculinity. Even if, as our above findings suggest, participants are indifferent to masculinity in “real life”, the application of “averageness” preferences to the faces used in masculinity research could generate systematic responses to particular faces, depending on their starting level of masculinity. Such a phenomenon would explain why morphing methods report directionally inconsistent effects of masculinity on attractiveness, with masculinised faces looking better approximately half of the time [57], [58].

Both of the more commonly used prior methods for assessing preferences may therefore elicit significant biases regarding masculinity with a frequency that offers apparent – but perhaps misleading – confirmation of its importance. To explore this possibility, we conducted a further study in which we re-measured masculinity preferences with both of these methods. That is, a) by assessing the relationship between a subjective measure of *perceived (i.e. rated)* masculinity and attractiveness, and b) by morphing each individual face to create a masculinised and a feminized version, then performing a two-alternative forced-choice (2AFC) preference test similar to those used in previous studies [59]. We used the same two sets of faces as in our previous experiment (section 2.2), for which we had already established that natural variation in morphological masculinity did not predict attractiveness. Based on prior results, we hypothesised that using these two prior methods would yield: a) a weak, positive correlation between rated masculinity and attractiveness, and b) a strong but directionally inconsistent effect of morphed masculinity on attractiveness, with feminine faces looking better masculinised and vice versa. Results such as these would show that prior methods produce statistically significant results *even when morphological masculinity does not really predict attractiveness*. If so, the use of such methods may have generated an inflated impression of the extent to which masculinity is a cue of attractiveness.

## Methods

All participants completed an informed consent form having been given written and verbal details of the tasks to be completed. This work was approved by the Faculty of Science Human Research Ethics Committee of the University of Bristol.

### Participants

#### Sample 1

One hundred and sixty-two women, recruited via University of Bristol. Twenty-two (age range 18–21, mean 19.5, SD .66), rated unmodified faces for attributes. A further 140 (age range 18–57, mean 20.33, SD 5.51) took part in the digital-morph forced-choice test (section 4.2.4).

#### Sample 2

One hundred and forty-eight students from Bristol and Stirling Universities. Eighteen (age range 19–41, mean 27, SD 7.34) rated faces on the attributes. A further 110 women (age range 18–70 mean 20.65, SD 4.94) performed the digital-morph forced-choice test.

### Stimuli

Original, unmodified photos from study 1 (see section 2.2) were used to test the influence of rated masculinity on attractiveness (see section 4.2.4). Masculinised and feminised versions of these same photographs were created and used as stimuli for the digital morphing approach, (see section 4.2.4).

### Rated masculinity

Photographs from both samples were rated for masculinity using an identical procedure to that in section 2.3.3 (see fig. 1 b) for examples). To investigate whether the relationship between rated masculinity and attractiveness is mediated by perceptions of health, ratings of apparent health were also collected. Inter-rater reliabilities were high for both variables (Sample 1: Cronbach's α = .902 (health), .829 (masculinity); sample 2:  = .904 (health), .880 (masculinity)).

### Preference for digitally masculinised versus feminised faces

#### Sample 1

Masculinised and feminised versions of the photographs were created using established software-based morphing techniques [29], [60]. That is: 168 x-y coordinates were used to define a male-female vector, determined by average differences in the position of the landmarks between male and female faces. The original 20 male images were transformed in shape along this vector, with each face transformed 80% in both masculine and feminine directions. This generated 20 pairs of masculinised and feminised male faces (see fig. 1 c).

Independent subjects then viewed these pairs of masculinised/feminised faces, in random order. Participants stated which of the two faces looked more attractive, on a scale of 1 to 8, following previous research [60]. The proportion of trials in which the masculinized face had been chosen was calculated for each face, to give an indication of how much a given face looked better masculinized.

#### Sample 2

Masculinised and feminised versions of the photographs were created using the same methods as in study 1, but with a 50% morph. The procedure was the same as for sample 1, and participant preferences were calculated for each face as before.

## Results

### Rated masculinity approach

In contrast to the results in section 3.1, there was a significant positive correlation between rated masculinity and attractiveness in both samples when using subjective measures (Pearson correlations, sample 1: r(20) = .513, p = .021, sample 2: r(75) = .248, p = .032, see table 2). This finding indicates that subjective judgments of masculinity are based on factors other than just morphological masculinity. Consistent with this, tests indicated that in both samples, rated masculinity was correlated with rated health (sample1: r(20) = .650, p = .002, sample 2: r(75) = .259 p<.025), and when rated health and rated masculinity were both entered into a regression as predictors of attractiveness, only rated health was a significant predictor of attractiveness (sample 1; health: Beta = .962, t = 6.723, p<.0005, masculinity: Beta = −.112, t = −.783, p = .444; sample 2; health: Beta = .779, t = 10.463, p<.0005, masculinity: Beta = .047, t = .625, p = .534). These results indicate that the impression of a relationship between masculinity and attractiveness, as reported in prior research using subjective measures of masculinity, may be an artefact of the way in which the term “masculinity” is interpreted by participants.

**Table 2:** Relationship between subjective traits and attractiveness.

	Association with Attractiveness^a^	
	*Sample 1 (n = 20)*	*Sample 2 (n = 75)*
Apparent healtha,b	.890***	.791***
Apparent masculinitya,b	.513*	.248*
Apparent masculinity, controlling for health^c^	−.112^ns^	.047^ns^

a As rated by participants.

b Result of Pearson correlation.

c Beta value when rated health is included in regression along with rated masculinity.

ns p>.10.

*p<.05.

**p<.01.

***p<.001.

### Digitally Morphed Masculinity Approach

To determine whether using masculinised and feminized versions of each face in a forced-choice design would elicit significant preferences for masculinity or femininity as predicted, a one-sample t-test was performed, with faces as subjects, on the proportion of trials for which the masculinised version of each face was chosen (against the chance value of 0.5). In sample 1, there was a significant overall bias towards masculinised versions being chosen (t(19) = 4.871, p<.0005). In sample 2, there was no significant overall bias in either direction, but this was largely due to directional preferences for certain faces cancelling each other out. A face-by-face analysis (binomial tests for deviations from 0.5) indicated that, in both samples, the majority of faces elicited significant directional preferences for either masculinity or femininity. In sample 1, 15 of the 20 (75.0%) faces elicited statistically significant directional preferences regarding masculinity/femininity, with 14 looking better masculinised. In sample 2, 40 of 75 (53.3%) the faces elicited significant directional preferences, with 22 looking better masculinised. Binomial tests indicated that for each sample the proportion of faces eliciting significant biases was significantly greater (p<.0001) than the 5% expected by chance if morphing did not tend to produce directional preferences for some faces; 75.0% for sample 1 (95% CI: 50.9%–91.3%) and 53.3% for sample 2 (95% CI: 41.5%–65.0%). These findings show that, by holding all other traits constant, digital morphing methods can elicit statistically significant preferences in a sample of photographs for which “real” variation in morphological masculinity does not actually predict attractiveness (as shown in section 3.1).

The existence of significant preferences in forced-choice experiments with morphed faces indicates that women agree on whether a given face looks better masculinised. As outlined in section 4.1, this fact is not inconsistent with “real life” indifference to masculinity, provided that some criteria can be identified (other than masculinity) via which women rank the test faces for attractiveness. Our results are somewhat consistent with the hypothesis that averageness is such a criteria: in the larger sample of photographs, level of morphological masculinity in the original photo was significantly and inversely related to the proportion of times that the masculinised version of a face was preferred by participants (r(75) = −.260, p<.026), although this effect was not statistically significant in sample 1 (r(20) = −.302, p = .196). That is, naturally masculine faces were more likely to look better feminised, and vice versa, suggesting that when participants were forced to discriminate on the basis of masculinity alone, “averagely” masculine faces were chosen. This finding provides an explanation as to how women can demonstrate a high level of agreement among themselves regarding whether a face looks better masculinised or feminised in forced choice experiments, in spite of being indifferent to masculinity in faces that vary naturally across multiple dimensions.

## Discussion

Most of the literature on facial masculinity preferences has focused on whether the influence of masculinity on attractiveness is positive or negative [57], [61]. Despite the volume of literature on facial preferences [9], no rigorous attempt has been made to affirm that masculinity matters when competing cues of health are available. In contradiction to the general assumption that masculinity does matter, our data provided no evidence of any systematic relationship, either linear or nonlinear, between masculinity and attractiveness in unmanipulated (i.e. real) faces. Instead, we find that yellow skin colour is a highly significant predictor of preferences, and explains more variation in attractiveness than either rated or measured masculinity. These results suggest that, for these samples at least, masculinity is not a primary determinant of male attractiveness.

There are a number of reasons to suppose that these findings are robust. Our stimuli depicted a wide range of faces, as the only dimension on which either sample was deliberately restricted was age (both samples depicted males of an appropriate age to be considered as possible mates by the participants). Indifference to masculinity was apparent in both group-level and individual level analyses, and patterns in perceptions were consistent across the two samples (tables 1, 2), with different pools of female raters, and with stimuli drawn from populations differing in geographic location and socio-economic status, and photographed under different lighting conditions. Our objective measure of masculinity was based on information from multiple feature-points, was a powerful discriminator between the sexes, and a significant predictor of attractiveness in female faces. If masculinity were a comparatively strong predictor of attractiveness in either sample, we would therefore expect to have detected this in our tests.

The indifference to morphological masculinity seen among our participants has not generally been apparent in prior research [although see 14], [ and 62 for similar findings relating to preference shifts]. Our proposed explanation for this fact is that two of the most common methods for measuring preferences produce significantly nonrandom results *even when masculinity doesn't matter*, and that these results have led to an overestimation of female interest in masculinity. Consistent with this proposal, we found that rated masculinity – in contrast to morphological masculinity – was a positive predictor of attractiveness, but this relationship was eliminated once health was controlled for. This finding indicates that participants' judgments when rating masculinity are based on additional traits other than morphological masculinity (such as colour cues, or potentially, semantic associations between masculinity and attractiveness) and hence explains the apparent attractiveness of masculinity in previous experiments. With regard to digital morphing methods, a significant majority of faces in both samples elicited directional preferences for either masculine or feminine versions, even though there was no relationship between “natural” morphological masculinity and attractiveness in either set of photos. These results show that preferences which are elicited via digital-morph forced-choice experiments are, potentially, a poor indicator of the real world importance of masculine face shape. Prior research has reported a relationship between responses to caricatured/forced-choice masculinity preferences tests and actual partner's masculinity [59], but the direction of causality is unclear. Our analyses suggest that responses in such experiments may reflect a general preference for averageness, rather than a particular interest in masculinity (although, we note that we did not find the predicted quadratic relationship between masculinity and attractiveness in experiment 1). Thus, partner's masculinity may influence perceptions of what is average and hence responses to caricatured stimuli, rather than preferences for masculinity influencing mate choice.

Nevertheless, our results require replication. If there is, in fact, any effect of masculinity on attractiveness, then this will be easier to detect in samples where variation in masculinity is high, and variation in other traits is low. The replication of our experiments in photosets with a wider range of ages, as well as a limited and/or attractive range of skin colours would therefore be instructive. In addition, we note that the individuals from our sample population have access to modern medicine and nutrition, high exposure to individuals from outgroups, and experience high levels of gender equality. Factors such as these may undermine the relationship between masculinity and immunity [8], [63] **,** introduce high levels of variation in the parameters of competing cues (such as colour, overall size, or other types of shape variation), or simply reduce interest in sex-typicality in general [64]. Interest in yellow skin colour might likewise be the result of some anomalous ecological or cultural factor, and it would therefore be instructive to attempt replication of our findings in populations with differing social and ecological backgrounds. The contrast between our participants' apparent lack of interest in sex-typicality and preferences observed in many non-human groups [1], also requires investigation. One possibility is that women do in fact find sex-typicality attractive, but rely on other cues such as body masculinity [47] or simpler cues of the type used in much non-human research, such as overall size [65]. Another, more speculative hypothesis, is that, following the logic of Adamo and Spiteri's model [22], humans are exposed to unusually fast levels of pathogenic fluctuation relative to life history, resulting in a reduced emphasis on stable cues of past disease resistance, and increased emphasis on condition.

In summary, our results suggest that the influence of masculine face shape on attractiveness may have been overstated in humans. This finding does not, of course, negate the possibility that masculinity may be a cue of other socially important traits such as age, dominance and/or aggression [19], [20], [29], and may therefore be of relevance both to personality perception and intrasexual selection. It does, however, contrast with the significant relationship between colour cues and attractiveness in these faces, and supports a shift in emphasis in physical attractiveness research toward the study of such condition-dependent cues.

## Supporting Information

Figure S1 The 129 facial landmarks used in the morphometric analyses of masculinity. Landmarks are represented on a composite female face. For definitions of landmarks see Stephan et al (2005).
(0.65 MB TIF)

Figure S2 Distribution of discriminant function scores for males and females, sample 1. Stacked histogram showing distribution of discriminant function scores for males (n = 31) and female (n = 31) from sample 1. Faces with discriminant scores >0 were classified as male by the function, those with scores <0 were classified as female.
(0.58 MB TIF)

Figure S3 Distribution of discriminant function scores for males and females, sample 2. Stacked histogram showing distribution of discriminant function scores for males (n = 75) and female (n = 75) from sample 2. Faces with discriminant scores >0 were classified as male by the function, those with scores <0 were classified as female.
(1.01 MB TIF)

Figure S4 Examples of skin patches from sample 2. a) Patches scoring low (left) and high (right) for lightness (L*) b) Patches scoring low (left) and high (right) for redness (a*) c) Patches scoring low (left) and high (right) for yellowness (b*)
(0.45 MB TIF)

Table S1 Principal components for the morphometric analysis of Sample 1.
(0.04 MB DOC)

Table S2 Principal components for the morphometric analysis of Sample 2.
(0.04 MB DOC)

## References

[1] Ryan MJ, Keddyhector A (1992). Directional Patterns of Female Mate Choice and the Role of Sensory Biases.. American Naturalist.

[2] Zahavi A (1975). Mate Selection - Selection for a Handicap.. Journal of Theoretical Biology.

[3] Hamilton WD, Zuk M (1982). Heritable True Fitness and Bright Birds - a Role for Parasites.. Science.

[4] Folstad I, Karter AJ (1992). Parasites, Bright Males, and the Immunocompetence Handicap.. American Naturalist.

[5] Angele MK, Schwacha MG, Ayala A, Chaudry IH (2000). Effect of gender and sex hormones on immune responses following shock.. Shock.

[6] Messingham KAN, Shirazi M, Duffner LA, Emanuele MA, Kovacs EJ (2001). Testosterone receptor blockade restores cellular immunity in male mice after burn injury.. Journal of Endocrinology.

[7] Muehlenbein MP, Bribiescas RG (2005). Testosterone-mediated immune functions and male life histories.. American Journal of Human Biology.

[8] Thornhill R, Gangestad SW (1999). Facial attractiveness.. Trends in Cognitive Sciences.

[9] Rhodes G (2006). The evolutionary psychology of facial beauty.. Annual Review of Psychology.

[10] Roberts ML, Buchanan KL, Evans MR (2004). Testing the immunocompetence handicap hypothesis: a review of the evidence.. Animal Behaviour.

[11] Pound N, Penton-Voak IS, Surridge AK (2009). Testosterone responses to competition in men are related to facial masculinity.. Proceedings of the Royal Society: Biological Sciences.

[12] Booth A, Johnson DR, Granger DA (1999). Testosterone and men's health.. Journal of Behavioral Medicine.

[13] Granger DA, Booth A, Johnson DR (2000). Human aggression and enumerative measures of immunity.. Psychosomatic Medicine.

[14] Thornhill R, Gangestad SW (2006). Facial sexual dimorphism, developmental stability, and susceptibility to disease in men and women.. Evolution and Human Behavior.

[15] Lie HC, Rhodes G, Simmons LW (2008). Genetic Diversity Revealed in Human Faces.. Evolution.

[16] Lassek WD, Gaulin SJC (2009). Costs and benefits of fat-free muscle mass in men: relationship to mating success, dietary requirements, and native immunity.. Evolution and Human Behavior.

[17] van Anders SM (2010). Gonadal Steroids and Salivary IgA in Healthy Young Women and Men.. American Journal of Human Biology.

[18] Rhodes G, Chan J, Zebrowitz LA, Simmons LW (2003). Does sexual dimorphism in human faces signal health?. Proceedings of the Royal Society of London Series B-Biological Sciences.

[19] Boothroyd LG, Jones BC, Burt DM, Cornwell RE, Little AC (2005). Facial masculinity is related to perceived age but not perceived health.. Evolution and Human Behavior.

[20] Scott I, Swami V, Josephson SC, Penton-Voak IS (2008). Context-dependent preferences for facial dimorphism in a rural Malaysian population.. Evolution and Human Behavior.

[21] Adamo SA, Spiteri RJ (2005). Female choice for male immunocompetence: when is it worth it?. Behavioral Ecology.

[22] Adamo SA, Spiteri RJ (2009). He's healthy, but will he survive the plague? Possible constraints on mate choice for disease resistance.. Animal Behaviour.

[23] Kalick SM, Zebrowitz LA, Langlois JH, Johnson RM (1998). Does human facial attractiveness honestly advertise health? Longitudinal data on an evolutionary question.. Psychological Science.

[24] Weeden J, Sabini J (2005). Physical attractiveness and health in western societies: A review.. Psychological Bulletin.

[25] Henderson JJA, Anglin JM (2003). Facial attractiveness predicts longevity.. Evolution and Human Behavior.

[26] Zhang D, Sonka M, Chen F, Zhang D (2010). A Benchmark for Geometric Facial Beauty Study..

[27] Komori M, Kawamura S, Ishihara S (2009). Effect of averageness and sexual dimorphism on the judgment of facial attractiveness.. Vision Research.

[28] Jones D, Hill K (1993). Criteria of Facial Attractiveness in 5 Populations.. Human Nature-an Interdisciplinary Biosocial Perspective.

[29] Perrett DI, Lee KJ, Penton-Voak I, Rowland D, Yoshikawa S (1998). Effects of sexual dimorphism on facial attractiveness.. Nature.

[30] Rhodes G, Hickford C, Jeffery L (2000). Sex-typicality and attractiveness: Are supermale and superfemale faces super-attractive.. British Journal of Psychology.

[31] Cunningham MR, Barbee AP, Pike CL (1990). What Do Women Want - Facialmetric Assessment of Multiple Motives in the Perception of Male Facial Physical Attractiveness.. Journal of Personality and Social Psychology.

[32] Koehler N, Rhodes G, Simmons L, Peters M (2003). Do symmetry and masculinity/femininity make independent contributions to attractiveness?. Australian Journal of Psychology.

[33] Neave N, Laing S, Fink B, Manning JT (2003). Second to fourth digit ratio, testosterone and perceived male dominance.. Proceedings of the Royal Society of London Series B-Biological Sciences.

[34] Rhodes G, Yoshikawa S, Palermo R, Simmons LW, Peters M (2007). Perceived health contributes to the attractiveness of facial symmetry, averageness, and sexual dimorphism.. Perception.

[35] Penton-Voak IS, Jones BC, Little AC, Baker S, Tiddeman B (2001). Symmetry, sexual dimorphism in facial proportions and male facial attractiveness.. Proceedings of the Royal Society of London Series B-Biological Sciences.

[36] Shackelford TK, Larsen RJ (1999). Facial attractiveness and physical health.. Evolution and Human Behavior.

[37] Jones BC, Little AC, Penton-Voak IS, Tiddeman BP, Burt DM (2001). Facial symmetry and judgements of apparent health - Support for a “good genes” explanation of the attractiveness-symmetry relationship.. Evolution and Human Behavior.

[38] Jones BC, Little AC, Burt DM, Perrett DI (2004). When facial attractiveness is only skin deep.. Perception.

[39] Zebrowitz LA, Rhodes G (2004). Sensitivity to “bad genes” and the anomalous face overgeneralization effect: Cue validity, cue utilization, and accuracy in judging intelligence and health.. Journal of Nonverbal Behavior.

[40] Fink B, Grammer K, Matts PJ (2006). Visible skin color distribution plays a role in the perception of age, attractiveness, and health in female faces.. Evolution and Human Behavior.

[41] Stephen I, Law Smith M, Stirrat M, Perrett D (2009). Facial Skin Coloration Affects Perceived Health of Human Faces.. International Journal of Primatology.

[42] Matts PJ, Fink B, Grammer K, Burquest M (2007). Color homogeneity and visual perception of age, health, and attractiveness of female facial skin.. Journal of the American Academy of Dermatology.

[43] Stephen ID, Coetzee V, Law Smith M, Perrett DI (2009). Skin Blood Perfusion and Oxygenation Colour Affect Perceived Human Health.. PLoS ONE.

[44] Russell R, Sinha P, Biederman I, Nederhouser M (2006). Is pigmentation important for face recognition? Evidence from contrast negation.. Perception.

[45] Hill H, Bruce V, Akamatsu S (1995). Perceiving the Sex and Race of Faces: The Role of Shape and Colour.. Proceedings: Biological Sciences.

[46] Tarr MJ, Kersten D, Cheng Y, Rossion B (2001). It's Pat! Sexing faces using only red and green.. Journal of Vision.

[47] Brown WM, Price ME, Kang J, Pound N, Zhao Y (2008). Fluctuating asymmetry and preferences for sex-typical bodily characteristics.. Proceedings of the National Academy of Sciences.

[48] Stephan CN, Penton-Voak IS, Perrett DI, Tiddeman BP, Clement JG, Marks M, Clement J (2005). Two-Dimensional Computer Generated Average Human Face Morphology and Facial Approximation.. Computer Graphic Facial Reconstruction: Elsevier Science.

[49] Tiddeman B, Burt DM, Perrett DI (2001). Prototyping and Transforming Facial Textures for Perception Research.. IEEE Computer Graphics and Applications.

[50] O'Higgins P, Jones N (1998). Facial growth in Cercocebus torquatus: an application of three-dimensional geometric morphometric techniques to the study of morphological variation.. Journal of Anatomy.

[51] Gower J (1975). Generalized procrustes analysis.. Psychometrika.

[52] Rohlf FJ, Slice D (1990). Extensions of the Procrustes Method for the Optimal Superimposition of Landmarks.. Syst Biol.

[53] Goodall C (1991). Procrustes Methods in the Statistical Analysis of Shape.. Journal of the Royal Statistical Society Series B (Methodological).

[54] Martinkauppi B (2002). Face color under varying illumination—analysis and applications..

[55] Frazier PA, Tix AP, Barron KE (2004). Testing moderator and mediator effects in counseling psychology research.. Journal of Counseling Psychology.

[56] Komori M, Kawamura S, Ishihara S (2009). Averageness or symmetry: Which is more important for facial attractiveness?. Acta Psychologica.

[57] DeBruine LM, Jones BC, Smith FG, Little AC (in press) Are attractive men's faces masculine or feminine? The importance of controlling confounds in face stimuli.. Journal of experimental psychology.

[58] Swaddle JP, Reierson GW (2002). Testosterone increases perceived dominance but not attractiveness in human males.. Proceedings of the Royal Society of London Series B-Biological Sciences.

[59] DeBruine LM, Jones BC, Little AC, Boothroyd LG, Perrett DI (2006). Correlated preferences for facial masculinity and ideal or actual partner's masculinity.. Proceedings of the Royal Society B-Biological Sciences.

[60] Jones BC, DeBruine LM, Little AC (2008). Adaptation reinforces preferences for correlates of attractive facial cues..

[61] Rennels JL, Bronstad PM, Langlois JH (2008). Are attractive men's faces masculine or feminine? The importance of type of facial stimuli.. Journal of Experimental Psychology-Human Perception and Performance.

[62] Peters M, Simmons LW, Rhodes G (2009). Preferences across the Menstrual Cycle for Masculinity and Symmetry in Photographs of Male Faces and Bodies.. Plos One.

[63] Daly M, Wilson MI (1999). Human evolutionary psychology and animal behaviour.. Animal Behaviour.

[64] Eagly AH, Wood W (1999). The origins of sex differences in human behavior - Evolved dispositions versus social roles.. American Psychologist.

[65] Pawlowski B, Koziel S (2002). The impact of traits offered in personal advertisements on response rates.. Evolution and Human Behavior.

