# Supplementary figures and images for: Does Masculinity Matter? The Contribution of Masculine Face Shape to Male Attractiveness in Humans
Source: PLoS One. 2010 Oct 27;5(10):e13585. doi: 10.1371/journal.pone.0013585 (PMC2965103; doi:10.1371/journal.pone.0013585)

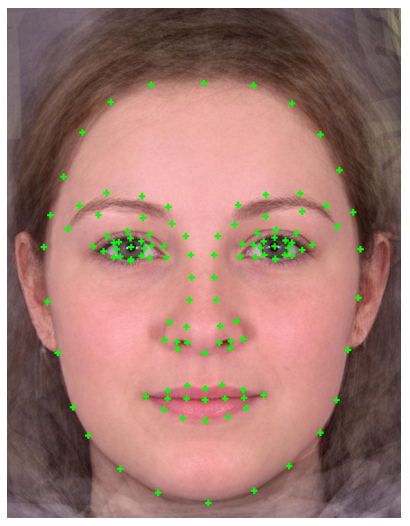

Supplement: Figure S1 — The 129 facial landmarks used in the morphometric analyses of masculinity. Landmarks are represented on a composite female face. For definitions of landmarks see Stephan et al (2005). (0.65 MB TIF) [file pone.0013585.s001.tif]

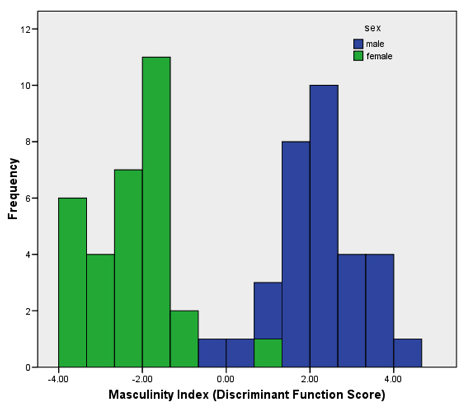

Supplement: Figure S2 — Distribution of discriminant function scores for males and females, sample 1. Stacked histogram showing distribution of discriminant function scores for males (n = 31) and female (n = 31) from sample 1. Faces with discriminant scores >0 were classified as male by the function, those with scores <0 were classified as female. (0.58 MB TIF) [file pone.0013585.s002.tif]

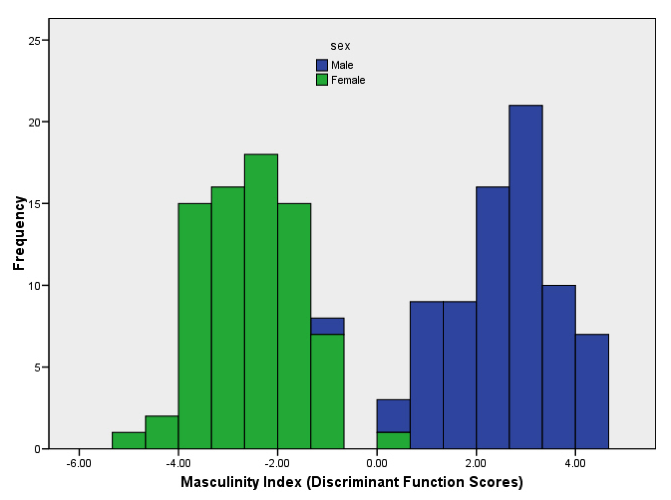

Supplement: Figure S3 — Distribution of discriminant function scores for males and females, sample 2. Stacked histogram showing distribution of discriminant function scores for males (n = 75) and female (n = 75) from sample 2. Faces with discriminant scores >0 were classified as male by the function, those with scores <0 were classified as female. (1.01 MB TIF) [file pone.0013585.s003.tif]

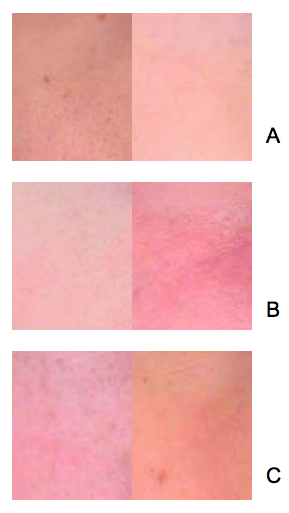

Supplement: Figure S4 — Examples of skin patches from sample 2. a) Patches scoring low (left) and high (right) for lightness (L*) b) Patches scoring low (left) and high (right) for redness (a*) c) Patches scoring low (left) and high (right) for yellowness (b*) (0.45 MB TIF) [file pone.0013585.s004.tif]
