# Supplementary material for: Does Masculinity Matter? The Contribution of Masculine Face Shape to Male Attractiveness in Humans
Source: PLoS One. 2010 Oct 27;5(10):e13585. doi: 10.1371/journal.pone.0013585 (PMC2965103; doi:10.1371/journal.pone.0013585)
Supplement: Table S1 — Principal components for the morphometric analysis of Sample 1. (0.04 MB DOC) [file pone.0013585.s005.doc]

Table S1 Principal components for the morphometric analysis of Sample 1.

| Principle Component | Eigenvalue† | Percentage individual variance | Percentage cumulative variance | Discriminant analysis results | |
| --- | --- | --- | --- | --- | --- |
|  |  |  |  | Partial lambda | Standardized discriminant function coefficients |
| 1 | 0.00175 | 28.5 | 28.5 | .847 | 0.974 |
| 2 | 0.00077 | 12.6 | 41.1 | .774 | -1.131 |
| 3 | 0.00066 | 10.7 | 51.8 | .777 | 1.126 |
| 4 | 0.00048 | 7.7 | 59.5 | .987 | 0.309 |
| 5 | 0.00044 | 7.2 | 66.7 | .915 | -0.756 |
| 6 | 0.00033 | 5.3 | 72.0 | .901 | -0.809 |
| 7 | 0.00027 | 4.3 | 76.3 | .985 | -0.333 |
| 8 | 0.00016 | 2.7 | 78.9 |  |  |
| 9 | 0.00014 | 2.2 | 81.1 |  |  |
| 10 | 0.00012 | 2.0 | 83.1 |  |  |
| 11 | 0.00010 | 1.7 | 84.7 | .977 | -0.407 |

PCs_ showing percentage variation, and eigenvalues for morphometric analysis of Sample 1. The first 11 PCs account for 84.7% of the variation in landmark configuration. Eight of these PCs (PC1-7 & PC11) were retained by a step-wise discriminant analysis which yielded a discriminant function (Wilks’ λ = 0.163; df = 8; χ2 = 101.6, p < 0.00001) that correctly classified 96.8% of male and 96.8% of female faces.

† Eigenvalues of the covariance matrix are used in the geometric morphometric analysis because the variables (Procrustes-registered landmark coordinates) have a common measurement scale. Sum of all eigenvalues equals the trace of the covariance matrix.
