# Supplementary material for: Does Masculinity Matter? The Contribution of Masculine Face Shape to Male Attractiveness in Humans
Source: PLoS One. 2010 Oct 27;5(10):e13585. doi: 10.1371/journal.pone.0013585 (PMC2965103; doi:10.1371/journal.pone.0013585)
Supplement: Table S2 — Principal components for the morphometric analysis of Sample 2. (0.04 MB DOC) [file pone.0013585.s006.doc]

Table S2. Principal components for the morphometric analysis of Sample 2.

| Principle Component | Eigenvalue | Percentage individual variance | Percentage cumulative variance | Discriminant analysis results | |
| --- | --- | --- | --- | --- | --- |
|  |  |  |  | Partial lambda | Standardized discriminant function coefficients |
| 1 | 0.00115 | 22.2 | 22.2 | .833 | -1.096 |
| 2 | 0.00058 | 11.1 | 33.3 | .431 | 1.455 |
| 3 | 0.00057 | 11.0 | 44.3 |  |  |
| 4 | 0.00039 | 7.5 | 51.8 | .981 | 0.401 |
| 5 | 0.00031 | 6.0 | 57.9 |  |  |
| 6 | 0.00022 | 4.2 | 62.0 | .988 | 0.317 |
| 7 | 0.00020 | 3.8 | 65.8 | .961 | 0.570 |
| 8 | 0.00014 | 2.6 | 68.4 | .993 | 0.243 |
| 9 | 0.00012 | 2.3 | 70.7 | .986 | -0.346 |
| 10 | 0.00011 | 2.1 | 72.8 |  |  |
| 11 | 0.00008 | 1.6 | 74.4 |  |  |
| 12 | 0.00008 | 1.6 | 76.0 |  |  |
| 13 | 0.00008 | 1.6 | 77.5 | .993 | 0.253 |
| 14 | 0.00007 | 1.4 | 78.9 | .994 | 0.229 |
| 15 | 0.00007 | 1.3 | 80.2 | .987 | 0.330 |
| 16 | 0.00005 | 1.1 | 81.3 |  |  |
| 17 | 0.00005 | 1.0 | 82.3 |  |  |
| 18 | 0.00005 | 0.9 | 83.2 |  |  |
| 19 | 0.00005 | 0.9 | 84.1 |  |  |
| 20 | 0.00004 | 0.8 | 84.9 |  |  |
| 21 | 0.00004 | 0.7 | 85.7 | .988 | 0.323 |

PCs_ showing percentage variation, and eigenvalues for morphometric analysis of Sample 2. The first 21 PCs account for 85.7% of the variation in landmark configuration. Eleven of these PCs (PC1, PC2, PC4, PC6-9, PC13-15 & PC21) were retained by a step-wise discriminant analysis which yielded a discriminant function (Wilks’ λ = 0.134; df = 11; χ2 = 286.6, p < 0.00001) that correctly classified 98.7% of male and 98.7% of female faces.
